# Association of usual sleep quality and glycemic control in type 2 diabetes in Japanese: A cross sectional study. Sleep and Food Registry in Kanagawa (SOREKA)

**DOI:** 10.1371/journal.pone.0191771

**Published:** 2018-01-24

**Authors:** Rika Sakamoto, Tadashi Yamakawa, Kenichiro Takahashi, Jun Suzuki, Minori Matsuura Shinoda, Kentaro Sakamaki, Hirosuke Danno, Hirohisa Tsuchiya, Manabu Waseda, Tatsuro Takano, Fuyuki Minagawa, Masahiko Takai, Tomohide Masutani, Jo Nagakura, Erina Shigematsu, Masashi Ishikawa, Shigeru Nakajima, Kazuaki Kadonosono, Yasuo Terauchi

**Affiliations:** 1 Department of Endocrinology and Diabetes Yokohama City University Medical Center, Yokohama, Japan; 2 Department of Biostatistics, School of Public Health, Graduate School of Medicine, The University of Tokyo, Tokyo, Japan; 3 Kanazawa Medical Clinic, Yokohama, Japan; 4 Department of Diabetes, Yokosuka City Hospital, Yokosuka, Japan; 5 Waseda Medical Clinic, Fujisawa, Japan; 6 Department of Diabetes and Endocrinology, Fujisawa City Hospital, Fujisawa, Japan; 7 Minagawa Medical Clinic, Yokohama, Japan; 8 Takai Medical Clinic, Kamakura, Japan; 9 Yata Ikeda Clinic, Mishima, Japan; 10 Department of Diabetes and Endocrinology, Yokohama Medical Center, Yokohama, Japan; 11 Ishikawa Medical Clinic, Yokohama, Japan; 12 Nakajima Medical Clinic, Yokosuka, Japan; 13 Department of Ophthalmology, Yokohama City University Medical Center, Yokohama, Japan; 14 Department of Endocrinology and Metabolism, Yokohama City University School of Medicine, Yokohama, Japan; Charite Medical University Berlin, GERMANY

## Abstract

**Objectives:**

Excessively short and long sleep durations are associated with type 2 diabetes, but there is limited information about the association between sleep quality and diabetes. Accordingly, the present study was performed to investigate this relationship.

**Materials and methods:**

The subjects were 3249 patients with type 2 diabetes aged 20 years or older. Sleep quality was assessed by using the Pittsburgh Sleep Quality Index (PSQI). A higher global PSQI score indicates worse sleep quality, and a global PSQI score >5 differentiates poor sleepers from good sleepers.

**Results:**

The mean global PSQI score was 5.94 ± 3.33, and 47.6% of the patients had a score of 6 or higher. Regarding the components of the PSQI, the score was highest for sleep duration, followed by subjective sleep quality and then sleep latency in decreasing order. When the patients were assigned to HbA1c quartiles (≤ 6.5%, 6.6–7.0%, 7.1–7.8%, and ≥ 7.9%), the top quartile had a significantly higher global PSQI score than the other quartiles. The top HbA1c quartile had a sleep duration of only 6.23 ± 1.42 hours, which was significantly shorter than in the other quartiles. Also, sleep latency was 25.3 ± 31.8 minutes in the top quartile, which was significantly longer (by approximately 20 minutes) than in the other quartiles. When analysis was performed with adjustment for age, gender, BMI, smoking, and other confounders, the global PSQI score was still significantly higher and sleep duration was shorter in the top HbA1c quartile (HbA1c ≥ 7.9%).

**Conclusions:**

Japanese patients with type 2 diabetes were found to have poor subjective sleep quality independently of potential confounders, especially those with inadequate glycemic control. Impairment of sleep quality was associated with both increased sleep latency and a shorter duration of sleep.

## Introduction

Both short and long sleep durations are associated with an increased risk of mortality, and a U-shaped association between sleep duration and mortality has been reported [1–4]. In recent years, U-shaped associations have been demonstrated between sleep duration and the risk of various lifestyle-related diseases, such as obesity [5], hypertension [6], coronary heart disease (CHD) [7], and atherosclerosis [8].

Poor sleep and insomnia with a short sleep duration are associated with an increased risk of diabetes [9–13]. In addition, emerging data have suggested the association of a long sleep duration with the risk of developing diabetes [11, 14]. Moreover, a number of epidemiological studies have revealed that difficulty with initiating or maintaining sleep is associated with a significantly increased risk of type 2 diabetes (T2DM) [14–17].

Sleep disorders are not only a risk factor for the onset of diabetes, but also have a great impact on patients with T2DM. Sleep disorders are more common in diabetic patients than in non-diabetic controls (33.7% vs. 8.2%; P < 0.01) [18]. The Pittsburgh Sleep Quality Index (PSQI) which is a self-rated questionnaire is widely used to assesses sleep quality [19]. More than half of all patients with diabetes are “poor sleepers” and patients with T2DM are more likely to have high scores on the PSQI [20]. Even when polysomnography data were adjusted for age, gender, and the apnea-hypopnea index, patients with diabetes and insomnia showed worse sleep efficiency than those without diabetes [21]. Furthermore, poor sleep was reported to be highly prevalent among T2DM patients and was inversely associated with the quality of life [22].

Moreover, the duration of sleep was reported to show an association with glycemic control in T2DM [23]. However, glycemic control may not only be affected by the quantity of sleep, but also by the quality of sleep. It was previously demonstrated that the majority of T2DM patients have poor quality sleep [24], and a relationship between poor sleep quality and higher glucose levels has been reported in diabetes [25–28]. However, most of these studies were performed in western countries and had a small samples size, while there have only been a few large-scale investigations of the sleep quality of T2DM patients in Asia, especially in Japan [29,30]. Therefore, the association between sleep quality and glycemic control remains unclear.

Accordingly, the aims of the present study were to investigate the quality of sleep among Japanese T2DM patients in Kanagawa who participated in the SOREKA study, and to assess the association between sleep quality and glycemic control.

## Materials and methods

### Subjects

The Sleep and Food Registry in Kanagawa is a multicenter prospective study designed to investigate the influence of modern treatment on the prognosis of patients with diabetes attending teaching hospitals certified by the Japan Diabetes Society or certified diabetes clinics in Kanagawa Prefecture, Japan (UMIN Clinical Trial Registry 000014318). Subjects were recruited from July 2014 to March 2016 at affiliated hospitals in Kanagawa Prefecture. A total of 4241 patients with diabetes or metabolic/endocrine diseases aged 20 years or older were registered for the baseline survey and were informed about the objectives of this study. Subjects were excluded if they fitted any of exclusion criteria 1) patients with drug-induced diabetes or steroid treatment; 2) patients with history of diabetic ketoacidosis or diabetic coma within 6 months prior to study entry; 3) patients receiving renal replacement therapy; 4) patients before or after surgery; 5) female patients who were pregnant or breast-feeding; 6) patients with other serious diseases in addition to diabetes, such as advanced malignancy, severe infection, severe trauma, and decompensated liver cirrhosis; and 7) other patients who were judged to be inappropriate for the study by the physicians, resulting in 3511 patients with T2DM being eligible. Among them, 137 patients were excluded because of failure to complete the questionnaire and 80 patients were excluded because of no HbA1c data, after which the remaining 3294 patients with T2DM were enrolled. Written informed consent was obtained from all participants. The PSQI questionnaire was performed and laboratory data were obtained within one month of enrollment. Each institutional ethics committees approved this study, which was performed in accordance with the Declaration of Helsinki. (Protocol: http://dx.doi.org/10.17504/protcols.io.irgcd3w)

### Clinical evaluation and laboratory measurements

Participants answered a questionnaire about demographic characteristics, medical history, and health-related habits at each annual examination. Sleep quality was measured by using the Pittsburgh Sleep Quality Index (PSQI), which is a validated self-rated questionnaire that assesses sleep quality and sleep disturbance over a 1-month period [19]. The 19 items in the index generate seven component scores that reflect problems in the following areas: subjective sleep quality, sleep latency, sleep duration, habitual sleep efficiency, sleep disturbances, use of sleep medications, and daytime dysfunction. The scores for these seven components are totaled to obtain a global score with a range of 0–21 points. A higher global PSQI score indicates worse sleep quality, and a global PSQI score > 5 has a diagnostic sensitivity of 89.6% and specificity of 86.5% for differentiating poor sleepers from good sleepers. The Japanese version of the PSQI has an overall reliability coefficient of 0.77 [31]. We assessed the global PSQI score and also the scores of the 7 components, which were determined by using a four-grade system (0, 1, 2, and 3) [19]. Subjective sleep quality was assigned a score of 0–3 points (0 = very good sleep quality; 1 = fairly good sleep quality; 2 = fairly bad sleep quality; and 3 = very bad sleep quality), as was sleep latency (time required to go to sleep each night and frequency of falling asleep within 30 minutes) and sleep duration (0 = sleep duration >7 hours; 1 = 6- ≤7 hours; 2 = 5-<6 hours; and 3 = <5 hours). Habitual sleep efficiency was also assigned a score of 0-3points (ratio of hours asleep to hours in bed: 0 = ≥85%; 1 = 75–84%; 2 = 65–74%; and 3 = <65%), as was sleep disturbance (trouble sleeping for some reason and the frequency). Furthermore, the use of sleep medication was assigned a score of 0-3points (0 = not used during the past month; 1 = used less than once a week; 2 = used once or twice a week; and 3 = used three or more times a week), as was daytime dysfunction (frequency of difficulty staying awake while driving, eating meals, or engaging in social activity and frequency of lacking enough enthusiasm to get things done).

Blood samples were collected from an antecubital vein. HbA1c was measured by high-performance liquid chromatography and the plasma glucose level was measured by the glucose oxidase method.

### Statistical analysis

All data were analyzed by cross-sectional manner. Results are reported as the mean ± SD, the median (25–75% inter-quartile range), or as numbers with percentages. Differences in the mean values and proportions of the characteristics of the subjects were assessed by ANOVA or the x2 test, as appropriate. Comparisons among 3 or more categories were performed with one-way ANOVA and the Tukey multiple comparison method. Univariate and multivariate linear regression analyses were performed to assess the relation between the PSQI global score and HbA1c or between sleep duration and HbA1c after adjustment for confounders. A two-sided p value <0.05 was considered to indicate statistical significance. All statistical analyses were performed using SPSS version 24.0 for Windows (IBM Corporation, NY, USA).

## Results

Baseline characteristics of the 3294 patients with T2DM are shown in Table 1. Their median age was 65 years (interquartile range: 55–72 years), median BMI was 24.6 kg/m^2^ (22.1–28.0 kg/m^2^), and median HbA1c was 7.1% (6.6–7.9%). These results suggested that the subjects were typical Japanese patients with T2DM [32]. Many patients had coexisting hypertension and/or dyslipidemia, and the prevalence of diabetic complications was higher than reported previously [33].

**Table 1:** Baseline characteristics.

n	3294
Age (years)	65 (55–72)
Gender (male/female)	2012 / 1282
Body mass index (kg/㎡)	24.6 (22.1–28.0)
Estimated duration (years)	11 (5–17)
Current smoker (%)	21.9
Alcohol (%)	53.1
Hypertension(%)	62.3
Dyslipidemia(%)	74.0
HbA1c(%)	7.1 (6.6–7.9)
Blood glucose(mg/dl)	143 (117–185)
Treatment—no medication(%)	11.1
Treatment—OHA only(%)	60.1
Treatment—Insulin or GLP-1(%)	28.8
Neuropathy(%)	43.3
Nephropathy(%)	12.4
Retinopathy(%)	23.6
Macro angiopathy(%)	19.5

Values were expressed as median (inter-quartile range, 25–75%) or the percentage.

Neuropathy: the presence of the subjective symptom (palsy, pain etc), or the weakening of Achilles tendon reflex, sense of vibration, or sensory nurve conduction velocity.

Nephropathy: Diabetic nephropathy stage 3, 4 or 5.

Retinopathy: simple, preproliferactive or proliferactive diabetic retinopathy.

Macro angiopathy: Ischemic heart disease, cerebral infarction, and arteriosclerosis obliterans

The value of blood glucose was for reference purpose because we did not fix previously when blood examination was checked in a day.

Tables 2, 3 and 4 display the results of the PSQI. The mean global PSQI score was 5.94 ± 3.33, and 47.6% of the patients had a score of 6 or higher, indicating poor sleep quality. Regarding the components of the PSQI, the score was highest for C3 (sleep duration), followed by C1 (subjective sleep quality) and then C2 (sleep latency) in decreasing order. The mean (± standard deviation) sleep duration was 6.40 ± 1.28 hours, mean sleep efficiency was 89.3 ± 12.3%, and mean sleep latency was 22.0 ± 24.3 minutes. When the patients were assigned to HbA1c quartiles (≤ 6.5%, 6.6–7.0%, 7.1–7.8%, and ≥ 7.9%; Table 2), the top quartile had significantly higher BMI and a higher percentage of current smokers. While there was no difference in the incidence of hypertension among the quartiles, dyslipidemia was more frequent in the top quartile. The prevalence of diabetic complications increased across the quartiles as HbA1 increased. Comparison of the PSQI data revealed that the global score was 5.6–5.8 in the 3 quartiles with an HbA1c ≤ 7.8%, and there was no significant difference of the global score among these quartiles. In contrast, the global PSQI score of the top quartile with the highest HbA1c (≥ 7.9%) was 6.50 ± 3.49, which was significantly higher than in the other quartiles. This quartile included a significantly larger percentage of patients with a global PSQI score ≥ 6 who were judged to have poor sleep quality. Regarding the components of the PSQI (C1–C7), the score was highest for C3, followed by C1 and then by C2 in all 4 quartiles. The top quartile (HbA1c ≥ 7.9%) had significantly higher scores for all of these components than the other 3 quartiles. There was no difference of sleep duration among the 3 lower quartiles with an HbA1c ≤ 7.8%, but the sleep duration of the top quartile (HbA1c ≥ 7.9%) was only 6.23 ± 1.42 hours and was significantly shorter than in the other quartiles. There were no differences of sleep efficiency among the quartiles. Sleep latency was approximately 20 minutes in the lower 3 quartiles (HbA1c ≤ 7.8%), and there was no difference of sleep latency among these quartiles as was the case for sleep duration. However, sleep latency was 25.3 ± 31.8 minutes in the top quartile (HbA1c ≥ 7.9%), and it was significantly longer than in the other quartiles.

**Table 2:** The clinical characteristics and the global PSQI score according to HbA1c.

	All	~6.5%	6.6~7.0%	7.1~7.8%	7.9%~	p
n	3294	820	795	824	855	
BMI (kg/㎡)	25.3±4.8	24.6±4.7	24.6±4.3	25.4±4.7	26.7±5.1	<0.001
Current Smoker (%)	21.9	21.6	19.7	19.2	28.3	<0.001
Alcohol (%)	53.1	54.4	55.1	49.9	53.6	0.159
Hypertension (%)	62.3	60.1	60.3	64.3	64.3	0.101
Dyslipidemia (%)	74.0	69.6	72.6	77.7	76.0	0.001
Neuropathy (%)	43.3	31.4	37.2	45.4	58.7	<0.001
Nephropathy (%)	12.4	10.1	10.7	14.2	14.4	0.010
Retinopathy (%)	23.6	17.1	19.1	25.4	32.1	<0.001
Macro angiopathy (%)	19.5	16.6	18.6	20.0	22.1	0.032
Global score	5.94 ±3.33	5.81 ±3.29	5.59 ±3.18	5.83 ±3.30	6.50±3.49	<0.001
Global score ≧6 (%)	47.6	46.3	43.9	46.6	53.4	0.001
C1	1.14 ±0.69	1.13 ±0.68	1.05 ±0.65	1.09± 0.65	1.27± 0.76	<0.001
C2	0.88± 0.93	0.86± 0.91	0.80± 0.88	0.87± 0.92	0.99± 1.00	<0.001
C3	1.51± 0.83	1.47± 0.81	1.47± 0.81	1.49± 0.83	1.59± 0.87	0.009
C4	0.62± 1.02	0.58± 0.98	0.58± 0.98	0.64± 1.04	0.66± 1.05	0.233
C5	0.84± 0.55	0.81± 0.56	0.80± 0.56	0.83± 0.53	0.91± 0.56	<0.001
C6	0.38± 0.96	0.46± 1.05	0.38± 0.94	0.35± 0.91	0.35± 0.93	0.050
C7	0.58± 0.71	0.51± 0.70	0.52± 0.68	0.54± 0.69	0.74± 0.76	<0.001
Sleep duration(hours)	6.40± 1.28	6.50± 1.18	6.46± 1.20	6.44± 1.30	6.23± 1.42	<0.001
Sleep efficiency(%)	89.3± 12.3	89.4± 11.9	89.9± 11.9	89.2± 12.9	88.6± 14.3	0.189
Sleep latency(minutes)	22.0± 24.3	21.1± 20.8	19.6± 18.7	21.5± 22.7	25.3± 31.8	<0.001

Values are expressed as means±SD or the percentage.

C1:subjective sleep quality, C2:sleep latency, C3:sleep duration, C4:habitual sleep efficiency, C5:sleep disturbance, C6:use of sleep medication, C7:daytime dysfunction

One-way ANOVA was performed. Comparison of Global score ≧ 6 was performed by x2 test.

**Table 3:** The distribution of C1-C7 according to HbA1c.

		All	~6.5%	6.6~7.0%	7.1~7.8%	7.9%~
C1	0	488 (14.8%)	121 (14.8%)	136 (17.1%)	123 (14.9%)	108 (12.6%)
	1	1961 (59.5%)	497 (60.6%)	491 (61.8%)	516 (62.6%)	457 (53.4%)
	2	742 (22.5%)	178 (21.7%)	157 (19.7%)	169 (20.5%)	237 (27.8%)
	3	104 (3.2%)	24 (2.9%)	11 (1.4%)	16 (1.9%)	53 (6.2%)
C2	0	1391 (42.2%)	349 (42.6%)	356 (44.8%)	354 (43.0%)	332 (38.8%)
	1	1151 (34.9%)	295 (36.0%)	285 (35.8%)	278 (33.7%)	292 (34.2%)
	2	501 (15.2%)	121 (14.8%)	109 (13.7%)	136 (16.5%)	135 (15.8%)
	3	252 (7.6%)	40 (4.9%)	45 (5.7%)	56 (6.8%)	96 (11.2%)
C3	0	503 (15.3%)	128 (15.6%)	122 (15.3%)	125 (15.2%)	128 (15.0%)
	1	852 (25.9%)	219 (26.7%)	220 (27.7%)	225 (27.3%)	188 (22.0%)
	2	1712 (52.0%)	433 (52.8%)	413 (51.9%)	417 (50.6%)	448 (52.5%)
	3	228 (6.9%)	40 (4.9%)	40 (5.0%)	57 (6.9%)	91 (10.6%)
C4	0	2379 (72.2%)	599 (73.0%)	585 (73.6%)	589 (71.5%)	606 (70.8%)
	1	3 (0.1%)	2 (0.2%)	0 (0.0%)	0 (0.0%)	1 (0.1%)
	2	713 (21.6%)	183 (22.3%)	172 (21.6%)	174 (21.1%)	183 (21.5%)
	3	200 (6.1%)	36 (4.4%)	38 (4.8%)	61 (7.4%)	65 (7.6%)
C5	0	795 (24.1%)	212 (25.9%)	215 (27.0%)	191 (23.2%)	177 (20.7%)
	1	2256 (68.5%)	559 (68.2%)	530 (66.7%)	581 (70.5%)	585 (68.5%)
	2	228 (6.9%)	44 (5.4%)	45 (5.7%)	49 (5.9%)	90 (10.5%)
	3	16 (0.5%)	5 (0.6%)	5 (0.6%)	3 (0.4%)	3 (0.4%)
C6	0	2809 (85.3%)	678 (82.7%)	674 (84.8%)	712 (86.4%)	744 (87.0%)
	1	62 (1.9%)	16 (2.0%)	23 (2.9%)	13 (1.6%)	10 (1.2%)
	2	74 (2.2%)	16 (2.0%)	18 (2.3%)	24 (2.9%)	16 (1.9%)
	3	350 (10.6%)	110 (13.4%)	80 (10.1%)	75 (9.1%)	85 (9.9%)
C7	0	1769 (53.7%)	489 (59.6%)	456 (57.4%)	457 (55.5%)	366 (42.9%)
	1	1198 (36.4%)	261 (31.8%)	272 (34.2%)	296 (35.9%)	369 (43.1%)
	2	276 (8.4%)	56 (6.8%)	59 (7.4%)	61 (7.4%)	100 (11.7%)
	3	52 (1.6%)	14 (1.7%)	8 (1.0%)	10 (1.2%)	20 (2.3%)

**Table 4:** Distribution of sleep duration according to HbA1c.

	All	~6.5%	6.6~7.0%	7.1~7.8%	7.9%~	p
n	3294	820	795	824	855	
<3 h	12	1	2	2	7	0.078
≥ 3 to <4 h	41	10	8	4	19	0.009
≥4 to <5 h	175	45	31	33	66	0.001
≥5 to <6 h	640	154	141	154	191	0.056
≥6 to <7 h	1067	261	289	259	258	0.194
≥7 to <8 h	856	227	223	218	188	0.015
≥8 to <9 h	381	91	95	102	93	0.578
≥9 to <10 h	85	23	27	16	19	0.354
≥10 h	37	11	5	7	14	0.192

### Correlate poor glycemic control

As shown in Table 5, multivariate analysis of factors influencing the global PSQI score revealed that the age, sex, BMI, and smoking were significant determinants of the score. The presence of diabetic neuropathy also affected the global score. When analysis was performed with adjustment for these factors, the global PSQI score of the top quartile (HbA1c ≥ 7.9%) was still significantly higher than the scores of the other 2 quartiles (6.6–7.0%, 7.1–7.8%). As shown in Table 6, sleep duration was influenced by age, sex, and the estimated duration of diabetes. Analysis with adjustment for these factors revealed that the sleep duration was likely to be shorter in patients from the top quartile (HbA1c ≥ 7.9%). Notably, the sleep duration of the top quartile was significantly shorter than that of the bottom quartile with the lowest HbA1c (≤ 6.5%).

**Table 5:** Univariate and multivariate linear regression analyses of clinical variables and the global PSQI score.

	Univariate	p	Multivariate	p
	β		β	
Age(year)	-0.032	<0.001	-0.018	0.001
Gender	0.634	<0.001	0.714	<0.001
Duration(years)	-0.013	0.097		
BMI(kg/㎡)	0.069	<0.001	0.037	0.005
Alcohol	-0.240	0.040	-0.062	0.624
Smoking	0.624	<0.001	0.596	<0.001
Hypertension	0.007	0.953		
Dyslipidemia	0.349	0.008	0.219	0.107
Neuropathy	0.356	0.003	0.370	0.003
Nephropathy	0.032	0.856		
Retinopathy	0.124	0.381		
Macro angiopathy	0.123	0.404		
HbA1c ~6.5%	-0.693	<0.001	-0.327	0.054
6.6~7.0%	-0.907	<0.001	-0.539	0.002
7.1~7.8%	-0.673	<0.001	-0.461	0.006
7.9%~	reference		reference	

**Table 6:** Univariate and multivariate linear regression analyses of clinical variables and sleep duration.

	Univariate	p	Multivariate	p
	β		β	
Age(year)	0.027	<0.001	0.021	<0.001
Gender	-0.154	0.001	-0.136	0.014
Duration(years)	0.020	<0.001	0.008	0.017
BMI(kg/㎡)	-0.031	<0.001	-0.009	0.137
Alcohol	0.036	0.429		
Smoking	-0.180	0.001	-0.053	0.417
Hypertension	0.120	0.009	0.015	0.805
Dyslipidemia	-0.073	0.150		
Neuropathy	0.048	0.294		
Nephropathy	0.116	0.090		
Retinopathy	0.079	0.145		
Macro angiopathy	0.230	<0.001	0.077	0.258
HbA1c ~6.5%	0.267	<0.001	0.168	0.024
6.6~7.0%	0.344	<0.001	0.137	0.071
7.1~7.8%	0.212	0.001	0.070	0.362
7.9%~	reference		reference	

Gender: male = 0, female = 1

Alcohol, Hypertension, Dyslipidemia, Neuropathy, Macro angiopathy: yes = 1, no = 0

Nephropathy (diabetic nephropathy stage 3,4 or 5): yes = 1, no = 0

Retinopathy (simple, preproliferative or proliferative diabetic retinopathy): yes = 1, no = 0

β: regression coefficient

## Discussion

In this study, the PSQI was used to examine the quality and quantity of sleep in 3294 Japanese patients with T2DM, revealing that their subjective sleep quality was poor and sleep duration was short. In particular, subjective sleep quality was worse in the patients with poor glycemic control (defined by an HbA1c ≥ 7.9%) and their sleep duration was also very short. The same findings were obtained by analysis with adjustment for confounders.

There have been several previous reports about the association between type 2 diabetes and sleep in Japanese patients. A study of 4870 Japanese patients with T2DM revealed a U-shaped association between sleep duration and the HbA1c level [23]. In addition, a study of 724 Japanese patients with T2DM and no history of cardiovascular disease demonstrated progression of arteriosclerosis in patients with poor sleep quality [34], suggesting a link between sleep and the development of diabetic complications. The amount of sleep that is necessary varies widely from person to person. A few studies have investigated factors such as sleep latency and sleep efficiency when examining how people assess their own sleep quality and whether or not these factors are linked to glycemic control, but hardly any large-scale studies of this kind have been carried out. Therefore, we examined the nature of sleep in Japanese patients with T2DM and assessed their subjective sleep quality and its relationship to HbA1c by using the PSQI, a questionnaire with verified reliability and validity that is designed to obtain information regarding the quality and quantity of sleep.

The mean sleep duration was 6.40 ± 1.28 hours. The 2011 Survey of Time Use and Leisure Activities performed by the Ministry of Internal Affairs and Communications demonstrated that Japanese people have an average sleep duration of 7 hours and 42 minutes[35]. In addition, the average sleep duration of people living in Kanagawa Prefecture was 7 hours and 31 minutes, which was the shortest reported among all 47 prefectures in Japan. Thus, the mean sleep duration of the patients with T2DM (6.40 ± 1.28 hours) was much shorter than that of the general population, even in Kanagawa Prefecture. The sleep duration of the top HbA1c quartile was significantly shorter than in the other quartiles, and this was compatible with a previous report [23]. Excessively short or long sleep duration has been reported to increase HbA1c and affect sleep quality [36]. The distribution of sleep duration in our subjects per HbA1c quartile is shown in Table 4, while the number of subjects (%) with each PSQI item is displayed in Table 3. In all 4 quartiles, almost the same percentage of subjects had a score of 0 points for C3 (= sleep duration > 7 hours). However, a significantly higher percentage of subjects with higher HbA1c levels had a score of 3 points (< 5 hours) (p < 0.001). Thus, a certain percentage of T2DM patients are long sleepers while a larger proportion of patients in the top quartile were found to be short sleepers in this study, which may explain the reduction of mean sleep duration.

In the present study, the mean global PSQI score of the T2DM patients was 5.94 ± 3.33 and 47.6% of them had a global score of 6 or higher. According to a study of the general population in Japan, 26.4% of males and 31.1% of females have a global PSQI score of 6 or higher [37], so our results suggest that T2DM patients have poor sleep quality.

We found that the global PSQI score was significantly higher in the top HbA1c quartile (≥ 7.9%) than in the other quartiles, and a significantly larger proportion of the patients this quartile had a global score of 6 or higher. When the scores for each component of the PSQI were compared, all 4 quartiles showed the highest score for C3 (sleep duration), followed by C1 (subjective sleep quality) and then C2 (sleep latency) in decreasing order. Although there were only a few study for T2DM considered in C1-C7 respectively of PSQI, Lou et al. reported that the highest PSQI score was observed for C2, followed by C5 and then C1 in decreasing order in a study of 944 patients with T2DM in China [22]. Thus, the results differed from those of the current study, which suggested that reduced sleep quality of Japanese patients with T2DM was mainly related to long sleep latency and short sleep duration. In practice, the quartile of patients with the highest HbA1c (≥ 7.9%) had significantly higher scores for C3, C1, and C2, the 3 components for which all 4 quartiles showed high scores in this study.

The present study revealed that the top quartile with the highest HbA1c level had a high global PSQI score, a short sleep duration, and a large BMI. The mechanisms underlying these findings may be as follows. First, a short duration of sleep causes an increase in the secretion of ghrelin, a hormone that increases appetite, and a decrease in the secretion of leptin, a hormone that suppresses appetite. Recently, another study showed that short sleep duration reduced gut hormones (PYY and GLP-1). These changes can increase food intake, which in turn may promote obesity and lead to the deterioration of glycemic control [5, 38]. Second, our study showed that the proportion of patients with diabetic complications increased along with an increase in HbA1c. Patients with high HbA1c levels tended to have more symptoms of hyperglycemia (such as thirst and nocturia) and more neuropathic pain [39], either of which could result in a short sleep duration and poor sleep quality. Third, reduction in the duration of sleep leads to elevation of the levels of cortisol, IL-6, and TNFα, resulting in activation of the sympathetic nervous system that promotes insulin resistance [40, 41]. Fourth, sleep deprivation can reduce QOL and interfere with daytime activities, as well as preventing patients from following their diet and exercise regimens. However, further investigations should be performed to confirm these findings and the underlying mechanisms.

The 2014 American Diabetes Association guidelines proposed that the glycemic control target for preventing microangiopathy should be to maintain an HbA1c level < 7.0%. If a patient has a history of severe hypoglycemia or has progression of complications, the target is to maintain an HbA1c < 8.0% [42]. In Japan, the 2013 Kumamoto Declaration of the Japan Diabetes Society also proposed that the glycemic control target for preventing complications is to maintain an HbA1c < 7.0%. If maintaining tight control is difficult due to factors such as hypoglycemia and adverse reactions, the glycemic control target is shifted to maintaining an HbA1c < 8.0%. It would also seem to be desirable to at least aim for an HbA1c level < 8.0% in order to prevent marked impairment of sleep quality. Conversely, interventions that improve sleep like treatment with hypnotic drugs might be expected to also improve glycemic control. The relation between use of hypnotic drugs to improve sleep and glucose tolerance has been examined in the past [43]. For example, suvorexant (an orexin receptor antagonist) was reported to improved glucose tolerance in a mouse model of T2DM [44]. If the same effect is observed in humans, it could be a promising treatment option for T2DM patients with sleep disorders. However, when ramelteon (a selective melatonin receptor agonist) was administered to 32 Japanese T2DM patients for 3 months, there was no change in HbA1c [45]. When the incidence of T2DM was examined in 45000 Taiwanese persons, patients using zolpidem were found to have a 45% higher risk of T2DM [46]. Accordingly, further studies on this issue seem to be required. There were several limitations of the present study. First, it was a cross-sectional study, rather than a prospective investigation. Second, the data were subjective, being based on answers to a questionnaire. Because we did not perform objective assessment using a method like polysomnography, the actual sleep duration and depth of sleep were not measured. Third, we were unable to examine the influence of sleep apnea syndrome [47] and restless leg syndrome [48], both of which are known to be common in patients with T2DM. A high proportion of patients with OSA or restless leg syndrome are poor sleepers with a PSQI >5 [49]. There have been reports that the majority of patients with sleep apnea syndrome are obese [50], and that a relation between AHI and HbA1c is observed in T2DM patients [51]. Since BMI was significantly higher in the top-quartile patients with HbA1c ≥ 7.9% in this study, many patients with OSA may have been included in that quartile. However, even when data obtained by polysomnography were adjusted for age, gender, and AHI, subjects with diabetes and insomnia had a lower sleep efficiency than those without diabetes [52]. In order to determine the possible influence of OSA or restless leg syndrome on our results, we are planning to perform polygraphy or polysomnography and the PSQI in T2DM patients in the future. Fourth, although the quality index (PSQI) that we used in the present study has been widely employed to assess sleep quality and was previously validated [19], it was reported that the PSQI score does not always accurately classify patients into good sleepers and poor sleepers, with a discrepancy between complaints about sleep and the actual results of PSG [53]. It was also reported that PSG reveals a decrease in total sleep duration and/or sleep efficiency in patients with insomnia, but this is not due to longer sleep latency and is mainly related to longer periods of wakefulness after sleep onset [54]. Ideally, sleep should not only be assessed by subjective measures such as the PSQI that we used, but also by objective methods. Finally, mental illnesses such as depression and schizophrenia show a higher prevalence in patients with diabetes [55, 56]. Although it is likely that such mental illnesses could contribute to sleep disorders, we were unable to screen patients for mental illness by detailed review of the medical records or identify symptoms of depression by using the Center for Epidemiologic Studies Depression Scale.

In conclusion, we investigated the pattern of sleep in 3294 Japanese patients with T2DM. We found that the patients had a shorter duration of sleep than the general population and poor subjective sleep quality, especially those with poor glycemic control characterized by an HbA1c ≥ 7.9%. Reduction of sleep quality was associated with both an increase in sleep latency and a shorter duration of sleep. Because diabetes and sleep seem to have interacting influences based on various factors, it is important for patients to get adequate sleep in order to support the management of diabetes.

## Supporting information

S1 File Data used for the analyses presented in this paper. (XLSX)
